# Associated Factors of Primary Cam Morphology in 4477 Early Adolescents: A Multiethnic, Population-Based, Cross-sectional Study (Generation R)

**DOI:** 10.1177/23259671251394027

**Published:** 2025-12-19

**Authors:** Delong Chen, Fleur Boel, Pim van Klij, Jos Runhaar, Fernando Rivadeneira, Sita M.A. Bierma-Zeinstra, Rintje Agricola

**Affiliations:** * Department of Orthopaedics and Sports Medicine, Erasmus MC University Medical Center Rotterdam, Rotterdam, the Netherlands; † The Generation R Study Group, Erasmus MC University Medical Center Rotterdam, Rotterdam, the Netherlands; ‡ Amsterdam UMC location University of Amsterdam, Department of Orthopedic Surgery and Sports Medicine, Academic Center for Evidence Based Medicine, Amsterdam IOC Center ACHSS, Amsterdam, the Netherlands; § Amsterdam Movement Sciences, Sports, Amsterdam, the Netherlands; ‖ Department of Sports Medicine, Isala Hospital, Zwolle, the Netherlands; ¶ Department of General Practice, Erasmus MC University Medical Center Rotterdam, Rotterdam, the Netherlands; # Department of Internal Medicine, Erasmus MC University Medical Center Rotterdam, Rotterdam, the Netherlands; Investigation performed at Erasmus MC University Medical Center, Rotterdam, the Netherlands

**Keywords:** primary cam morphology, hip osteoarthritis, associated factors, early adolescents, general population

## Abstract

**Background:**

Multiple prospective cohort studies have consistently shown a strong association between cam morphology and the development of hip osteoarthritis. However, associated factors of primary cam morphology (PCM) in the general adolescent population remain largely unexplored.

**Purpose:**

To investigate associated factors of the presence of PCM and increased alpha angle in early adolescents from the general population.

**Study Design:**

Cross-sectional study; Level of evidence, 3.

**Methods:**

The authors included 4477 participants with high-resolution dual-energy x-ray absorptiometry scans of the right hip from the population-based Generation R cohort in Rotterdam, the Netherlands. The alpha angle was automatically measured using validated methods. PCM was defined as an alpha angle ≥60°. The authors used multivariable logistic and linear regression models to investigate factors (demographic, anthropometric, and lifestyle factors and proximal femoral physis status) associated with PCM and increased alpha angle, respectively.

**Results:**

Of the included participants (median age, 13.5 years [2.5th-97.5th percentile, 13.2-14.6]; 51.6% female, 73.7% European), 151 (3.4%) had PCM. Male sex (adjusted odds ratio [aOR], 2.56; 95% CI, 1.62-4.04) and closed proximal femoral physis (aOR 3.28; 95% CI, 2.05-5.24) were associated with higher odds of PCM, whereas underweight (aOR 0.42; 95% CI, 0.18-0.97) was associated with lower odds of PCM. Factors associated with increased alpha angle included male sex (β coefficient, 2.99; 95% CI, 2.40-3.58), closed proximal femoral physis (β coefficient, 1.22; 95% CI, 0.61-1.82), cutting sport type (β coefficient, 1.07; 95% CI, 0.30-1.85), overweight (β coefficient, 0.89; 95% CI, 0.22-1.55), and obesity (β coefficient, 1.81; 95% CI, 0.57-3.04). Ethnicity and physical activity frequency were not associated with PCM or increased alpha angle.

**Conclusion:**

Among Dutch early adolescents, male sex and proximal femoral physis closure were associated factors of PCM and increased alpha angle, while cutting sport type, overweight, and obesity were modifiable factors of increased alpha angle. These findings could inform potential primary prevention strategies for PCM.

Osteoarthritis is a common and chronic degenerative disease driven by mechanical, inflammatory, and metabolic factors that is getting increasing attention owing to its patient burden.^24,25^ Multiple risk factors predispose individuals to hip osteoarthritis, including genetic predisposition, high-impact sports, high occupational loading, and altered hip morphologies.^51^ Growing evidence suggests that hip morphology plays an important role in the development of hip osteoarthritis.^2,39,41,45,47^ One of the bony hip morphologies associated with hip osteoarthritis is cam morphology, which refers to an aspherical femoral head with a bony prominence at the femoral head-neck junction.^12^ This bony prominence is most commonly evaluated by the alpha angle in different imaging modalities and planes.^12^ When symptoms and clinical signs coexist, it is known as *femoroacetabular impingement syndrome* (FAIS).^20^ Cam morphology can lead to intra-articular damage^8,21^ when repetitively forced into the acetabulum during hip motion, predisposing some individuals to FAIS and eventually to the development of hip osteoarthritis later in life.

*Primary cam morphology* (PCM) refers to cam morphology that develops as an adaptive bone response to mechanical loading during various athletic activities in adolescence.^12^ Small studies in male athletes reported that it gradually develops from 12 to 13 years onward,^1,37^ but its cause in early adolescence is not completely understood. In adults, some cross-sectional studies have reported that a higher prevalence of PCM was present in male athletes, particularly in cutting and impingement sports, such as ice hockey,^30,38^ soccer,^18,27,29^ and basketball.^43^ A dose-response relationship between sporting activity and PCM development has been observed in some studies.^16,37,44^ A small retrospective study found that male elite soccer players who trained at least 4 times per week before age 12 years were at greater risk of PCM.^44^ A cross-sectional study by Palmer etal^37^ demonstrated that adolescent elite soccer athletes had significantly higher alpha angles than amateur athletes and nonathletes. Fernquest etal^16^ confirmed this dose-response relationship in a 3-year longitudinal follow-up, showing that adolescents with higher physical activity levels experienced greater increases in alpha angles.

In addition to physical loading from sports participation, ethnicity may be another associated factor. A cross-sectional study involving 445 elite male soccer players found that East Asian players had a lower prevalence of PCM as compared with other ethnicities.^33^ These ethnic differences may reflect underlying genetic predispositions. However, the current research on the cause of PCM is predominantly focused on male athletes, with small sample sizes. Associated factors of PCM in the general adolescent population remain largely unexplored. Moreover, there is a lack of epidemiologic studies evaluating the cause of PCM in females and among multiethnic populations, which aligns with the priorities set by the latest Oxford consensus.^14^ Understanding modifiable factors of PCM during skeletal maturity may offer a window for primary prevention strategies of PCM before it leads to hip osteoarthritis later in life.

The first aim of our study was to determine factors associated with PCM in early adolescents from the general population. Our second aim was to determine factors associated with increased alpha angle.

## Methods

### Study Design and Participants

We performed a cross-sectional analysis within the Generation R cohort, an ongoing multiethnic, population-based, prospective cohort in Rotterdam, the Netherlands, which followed participants from fetal life onward. The study was approved by the Medical Ethical Committee of the Erasmus University Medical Center (MEC-2015-749), and informed consent was obtained from all participating adolescents and their parents. More detailed information on the Generation R cohort is available elsewhere.^28^ In total, 4929 participants attended the visit around the age of 13 years (Focus 13), of whom those with dual-energy x-ray absorptiometry (DXA) scans of their right hips were included in this study. We excluded participants if they lacked alpha angle measurements attributed to incomplete depiction of the femoral head or neck, movement artifacts, and artifacts in the region of interest. This study followed the STROBE (Strengthening the Reporting of Observational Studies in Epidemiology) reporting guideline.^50^

### DXA Scans

In studies involving healthy childhood or adolescent populations where minimizing radiation exposure is a priority, a DXA scan offers a lower-radiation alternative for hip morphology assessment than radiographs. The anteroposterior DXA scans of the right hip of all participants were performed by well-trained investigators using a General Electric Lunar iDXA densitometer (iDXA Scanner; GE Healthcare). The participants were positioned supine on the scanning table with their legs slightly separated and internally rotated and their big toes touching. The feet were secured in this position by a Velcro strip.^7^

### Measurements of Alpha Angle

We defined PCM as an alpha angle ≥60°.^4,7,49^ To increase statistical power to detect associations, we also used the alpha angle as a continuous outcome, aligning with recommendations from the Oxford consensus that emphasize using continuous measures for research on PCM etiology.^13^

**Figure 1.**
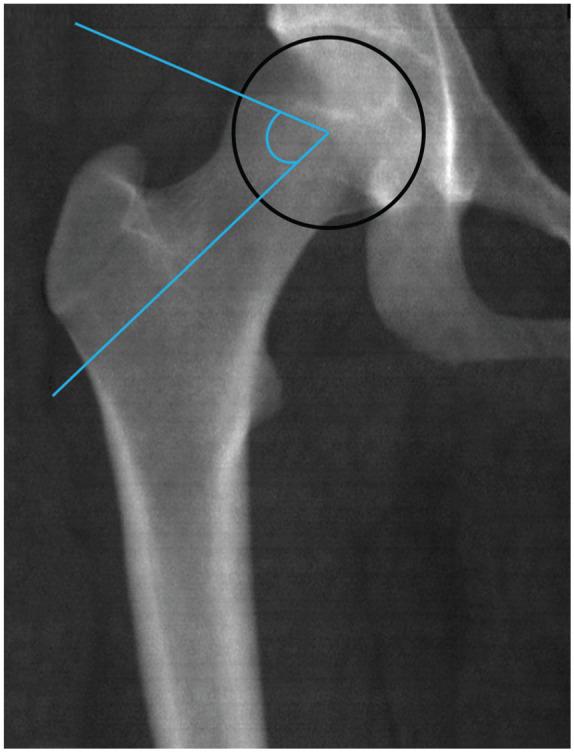
Measurement of alpha angle on a dual-energy x-ray absorptiometry image of the right hip.The alpha angle is formed by the intersection of 2 lines. From the best-fitting circle center around the femoral head, one line extends to the point where the femoral head first departs from the margin of the best-fitting circle, and the second line extends to the femoral neck center.

The alpha angle was automatically measured by using landmarks in open-access validated software developed in-house.^5^ These landmarks outlining the bony contour of the hip were automatically placed on the right hip DXA images using BoneFinder software^31^ (www.bone-finder.com; The University of Manchester). The alpha angle quantifies the deviation of the femoral head from sphericity and is formed between 2 lines (Figure 1). The first line connects the center of the best-fitting circle around the femoral head to the point where the femoral head first departs the margin of the circle; the second line connects the center of the best-fitting circle to the center of the femoral neck. Detailed methods of measurement were described by Boel etal,^5^ and good intermethod reliability (0.81; 95% CI, 0.46-0.92) was observed between manual measurement and the automated method. Furthermore, recent research has demonstrated that alpha angle measurements performed on supine DXA images are comparable to those on weightbearing pelvic radiographs in adults, with good reliability (0.72; 95% CI, 0.68-0.75) between the imaging modalities.^6^

### Associated Factors

#### Demographic Factors

The participant's sex assigned at birth was obtained from hospital registries and medical records. The age of participants was calculated at the date of DXA scans. According to the official definition of the Dutch Central Bureau of Statistics, the ethnicity of participants was determined by the country of birth of their parents, on which information was collected from questionnaires. We categorized the ethnicity of participants into 4 groups: European (Dutch, Turkish, European, Oceanian, and American Western [including North American]), African (Cape Verdian, Moroccan, Dutch Antilles, Surinamese-Creole, and other African), Asian (Indonesia, Surinamese-Hindustani, other Asian), and other (Surinamese unspecified and American non-Western [including South American and Central American]).^10,26^

#### Proximal Femoral Physis Status

The skeletal maturity of the femoral head was assessed by 1 investigator (D.C.) and was determined by the status of the proximal femoral physis (open or closed) on the right hip DXA images. Complete fusion of the physis indicates skeletal maturity, while an open physis indicates skeletal immaturity. The intra- and interobserver reliabilities of the proximal femoral physis evaluation were 0.90 (95% CI, 0.85-0.94) and 0.87 (95% CI, 0.78-0.92), respectively.^7^

#### Anthropometric Factor

The body mass index (BMI; kilograms/meters squared) was used to categorize the weight status of adolescents based on their height and weight, which were measured at the research center. Based on the age- and sex-specific BMI cutoffs proposed by the International Obesity Task Force,^9^ adolescents’ weight was categorized into 4 groups: underweight, normal weight, overweight, and obesity. For the median age of our study population (13.5 years), the task force's specific BMI cutoffs were as follows: underweight (<16.11 for males and <16.55 for females), overweight (>22.24 and >22.90), and obesity (>27.26 and >28.03).

#### Lifestyle Factors

Detailed information on sport types and physical activity frequency was obtained by self-reported questionnaires. Sport was categorized into 7 groups based on the mechanical loading placed on the hip^22,34^: cutting, flexibility, asymmetric, impingement, endurance, contact, or other sports (Table 1). For participants involved in ≥2 sports, the sport with the most time spent was considered the primary sport, which was then categorized into 1 of the 7 groups. Participants were grouped into mixed sports if equal time was spent across multiple sport categories. Given the limited sample size, contact sport type (n = 21) was grouped into cutting sport type because they share similar movements involving sudden directional changes during sporting activity. We determined the frequency of the physical activity based on the number of days per week that participants were physically active at least 1 hour, and we dichotomized it into 2 categories: high frequency (≥4 days per week) and low frequency (<4 days per week).^44^

**Table 1:** Sport Categories Based on Mechanical Loading on the Hip

Category	Sports
Cutting	Soccer, hockey, basketball, korfball, handball, skiing, surfing, skateboarding, longboarding
Flexibility	Dancing, judo/(kick)boxing/martial arts, gymnastics, free running, rock climbing, synchronized swimming, figure skating, yoga, combat sport
Asymmetric	Baseball, softball, tennis, volleyball, badminton, table tennis, cricket, golf, squash, sword fighting
Impingement	Horseback riding, water polo, rowing, CrossFit, skating, ice hockey, weight lifting, gym, fitness
Endurance	Athletics, running, swimming, cycling/mountain biking
Contact	Rugby, football
Other	Sailing, archery, diving, darts, scootering, karting

### Statistical Analyses

We used multivariable logistic and linear regression models to examine factors associated with PCM and increased alpha angle in the general population. The reference groups selected for each categorical variable were female sex, European ethnicity, open proximal femoral physis, normal weight, low physical activity frequency, and no participation in sports. Effect estimates were reported as adjusted odds ratios (aORs) for binary outcomes or adjusted β coefficients for continuous outcomes with corresponding 95% confidence intervals, which were visualized with forest plots. We examined linear regression model assumptions (normality, homoscedasticity, and multicollinearity) and did not find violations.

Missing data for ethnicity (2.2% missing), sport type (17.7% missing), and physical activity frequency (31.7% missing) were assumed to be missing at random. To maintain statistical power and minimize potential bias from missing data, we handled missing data using the MICE (multiple imputations by chained equations) method in which 40 data sets with 30 iterations were created, and we reported the pooled results. We found no major differences in participant characteristics between the observed and imputed data sets (Appendix Table A1). To assess the robustness of our findings, we conducted 2 sensitivity analyses. First, we repeated analyses in the complete cases data set to evaluate whether missing data patterns influence the primary findings. Second, we excluded mixed sports from our analysis to assess the effect of a single sport type on PCM. We conducted statistical analyses using R Statistical Software (Version 4.2.1; R Core Team), constructed forest plots using “forest plot” package,^19^ and performed multiple imputations using “mice” package.^46^

## Results

### Participant Characteristics

**Figure 2.**
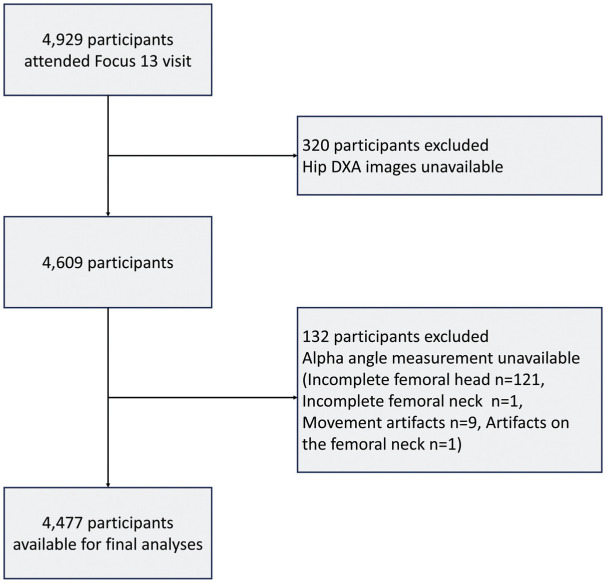
Flowchart of participants included for analyses. DXA, dual-energy x-ray absorptiometry.

Figure 2 shows the flowchart of participants. A total of 4477 participants were included in the current analyses. Table 2 shows the characteristics of the included participants. The median alpha angle was 43.4° (2.5th-97.5th percentile, 35.4°-64.0°). The overall prevalence of PCM was 3.4% (n = 151; 84 males and 67 females) in early adolescents.^7^

**Table 2:** Characteristics of the Study Population (4477 Adolescents)*^*a*^*

Characteristic	No. (%)
Age, y	13.5 (13.2-14.6)*^*b*^*
Sex	
Female	2311 (51.6)
Male	2166 (48.4)
Ethnicity	
European	3368 (75.2)
African	713 (15.9)
Asian	264 (5.9)
Other	132 (2.9)
Proximal femoral physis status	
Open	2626 (58.7)
Closed	1851 (41.3)
Weight categories	
Underweight	468 (10.4)
Normal weight	3280 (73.3)
Overweight	578 (12.9)
Obesity	151 (3.4)
Physical activity frequency	
Low	1526 (34.1)
High	2951 (65.9)
Sport type	
None	761 (17.0)
Cutting	1720 (38.4)
Flexibility	587 (13.1)
Asymmetric	274 (6.1)
Impingement	276 (6.2)
Endurance	284 (6.4)
Other	41 (0.9)
Mixed	534 (11.9)
Primary cam morphology	151 (3.4)
Alpha angle, deg	43.4 (35.4-64.0)*^*b*^*

a Percentages of missing values before multiple imputation: 2.2% (n = 100) for ethnicity, 17.7% (n = 792) for sport types, and 31.7% (n = 1419) for physical activity frequency.

b Median (2.5th-97.5th percentile).

### Associated Factors of PCM

**Figure 3.**
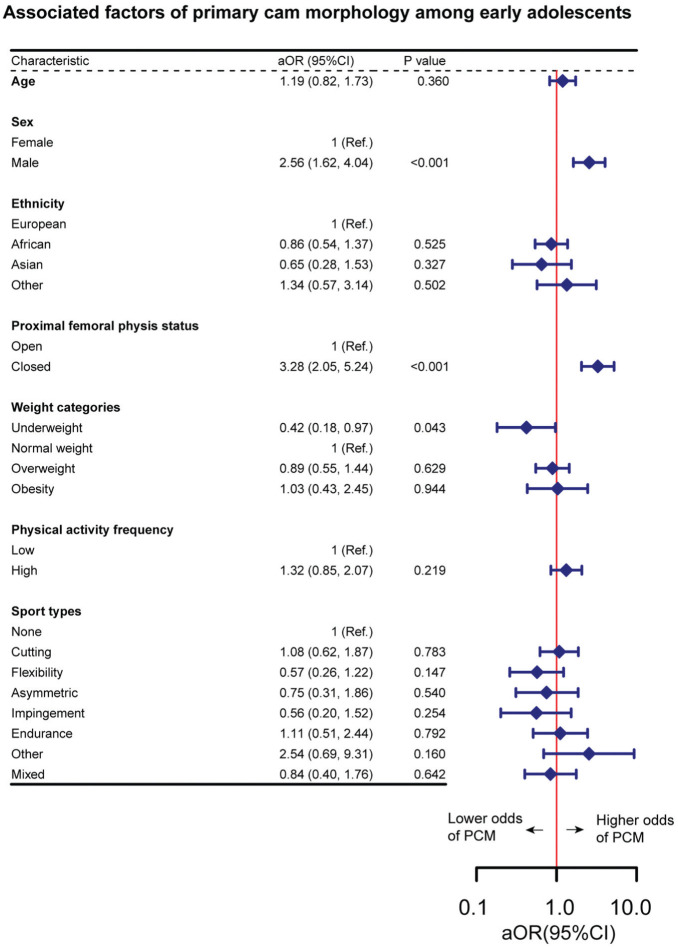
Associated factors of primary cam morphology among early adolescents. The multivariable logistic regression analysis was based on the imputed data set and was the pooled result of 40 data sets. aOR, adjusted odds ratio; PCM, primary cam morphology.

In the multivariable logistic regression analysis model, male sex (aOR, 2.56; 95% CI, 1.62-4.04) and closed proximal femoral physis (aOR, 3.28; 95% CI, 2.05-5.24) were associated with higher odds of PCM, and underweight (aOR, 0.42; 95% CI, 0.18-0.97) was associated with lower odds of PCM in this study population (Figure 3).

### Associated Factors of Increased Alpha Angle

**Figure 4.**
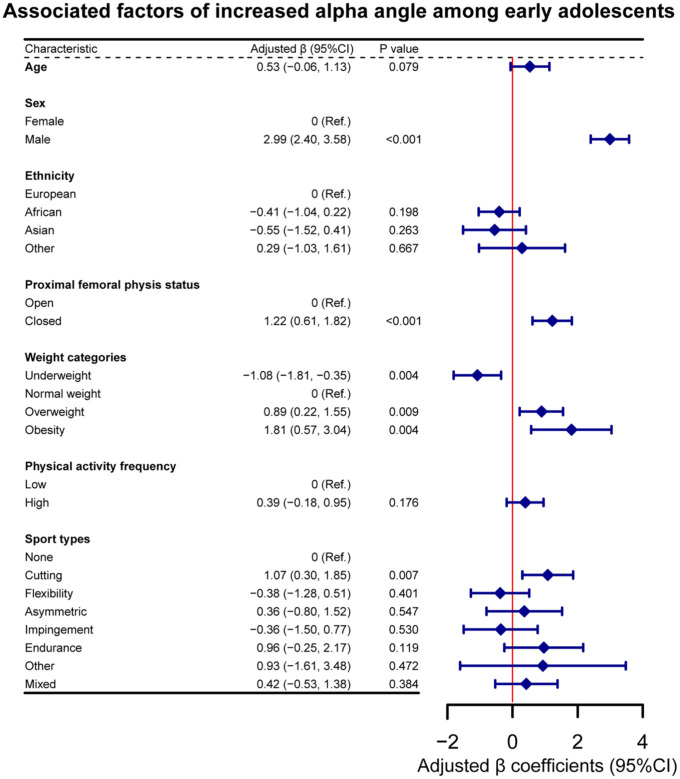
Associated factors of increased alpha angle among early adolescents. The multivariable linear regression analysis was based on the imputed data set and was the pooled result of 40 data sets.

In the multivariable linear regression model, male adolescents had an alpha angle that was on average 2.99° (95% CI, 2.40°-3.58°; *P* < .001) higher than female adolescents (Figure 4). Participants with a closed proximal femoral physis had an alpha angle that was 1.22° (95% CI, 0.61-1.82; *P* < .001) higher than those with an open proximal femoral physis. When compared with adolescents with normal weight, those with underweight had an alpha angle that was 1.08° (95% CI, –1.81° to −0.35°) lower, and those with overweight and those with obesity had an alpha angle that was 0.89° (95% CI, 0.22°-1.55°; *P* = .009) and 1.81° (95% CI, 0.57°-3.04°; *P* = .004) higher, respectively. Adolescents who played cutting sports had an alpha angle that was 1.07° (95% CI, 0.30°-1.85°; *P* = .007) higher than those who did not play sports.

### Sensitivity Analyses

When we repeated the analyses using the complete cases data set (n = 2874), we observed similar directions and strengths of the effect estimates in most variables (Appendix Tables A2 and A3). However, the associations of increased alpha angle with overweight and obesity were attenuated and became statistically nonsignificant. When we excluded mixed sports from our analyses (Appendix Tables A4 and A5), the association between overweight and increased alpha angle became statistically nonsignificant, while the association for obesity remained significant.

## Discussion

In this multiethnic population-based study, we observed that male sex and closed proximal femoral physis were associated with higher odds, and underweight with lower odds, of PCM in early adolescents from the general population. In addition, male sex, closed proximal femoral physis, cutting sport type, overweight, and obesity were associated with an increased alpha angle.

Owing to its high prevalence, PCM is frequently studied within male athletic populations.^32,35,52^ In adults, Doran etal^15^ compared the prevalence of PCM across sport types in a systematic review and suggested that athletes participating in cutting, impingement, and contact sports had a higher prevalence of PCM. Moreover, Philippon etal^38^ found that male ice hockey players were 4.5 times more likely to develop PCM than skiers. This indicated that specific sport types and hip loading patterns significantly influence the development of PCM. We observed an association between increased alpha angle and cutting sport type in early adolescents from the general population, which might be primarily driven by the high rotational and shear forces exerted on the proximal femoral physis during rapid changes of direction. A finite element analysis revealed that there is a trigger for extra bone formation at the anterolateral head-neck junction when loading patterns of external rotation and flexion are applied to a hip with an open proximal femoral physis.^40^

There is variation in the reported relationship between body weight and alpha angle in the literature, seemingly depending on the study population. In this study, overweight and obesity were associated with increased alpha angle in early adolescents from the general population in the main analysis, but these associations became nonsignificant in the sensitivity analysis of the complete cases. These findings were supported by Novais etal,^36^ who found that a higher BMI percentile was associated with an increased alpha angle in a retrospective study based on pelvic computed tomography from 128 male and female adolescents aged 12 to 18 years without hip complaints. The mechanism of association between higher weight categories and increased alpha angle is unclear and may involve multiple factors, such as altered metabolic and hormonal profiles and increased mechanical loading.^42^ However, some cross-sectional and longitudinal studies have found no association between BMI and increased alpha angle in male athletes.^16,37^ This discrepancy could be attributed to the different loading patterns and physical demands between the athletic and general populations. Athletes experience high-impact and repetitive loading on their hips attributed to sport-specific movements (eg, hip flexion and rotation), which may lead to the development of PCM independent of BMI. In contrast, the general population experienced less sport-specific hip loading and may be more influenced by overall body weight. The sensitivity analysis in our study showed that the relationship between alpha angle and overweight and obesity became nonsignificant when the sample size decreased, suggesting that this association might be weak. Therefore, larger cohort studies are needed to validate this association across different demographic groups.

Some small prospective studies in male athletes have shown that PCM gradually increases until proximal femoral physis closure.^3,48^ A cross-sectional study of 210 individuals (103 male soccer players and 107 controls) aged 9 to 18 years found that individuals with a closed proximal femoral physis exhibited a maximum cartilage alpha angle 13° higher than those with an open proximal femoral physis on magnetic resonance imaging.^37^ Fernquest etal^16^ performed a 3-year longitudinal study of 140 individuals (69 male soccer players and 71 controls) and observed no significant increase in alpha angle in individuals with a closed proximal femoral physis at baseline. Similarly, our study suggested that skeletal maturity of the femoral head (closed proximal femoral physis) was associated with the presence of PCM and increased alpha angle in the general population. This implies that the process of femoral head skeletal maturity is a critical window during which adolescents may be at increased risk for the development of PCM. In this study, the participants with an open proximal femoral physis still have the potential to develop PCM when they become older. The timing of potential primary prevention strategies for PCM in the general population should therefore precede the closure of the proximal femoral physis.

To our knowledge, this is the first study to identify associated factors of PCM in the general population. The presence of PCM is common in the general population^11,17^ and is known to be a strong and potentially modifiable risk factor for hip osteoarthritis. Our results are of etiologic and potentially preventive interest, but they may not directly be applied to clinical settings, where patients typically present because of symptoms. Primary prevention of PCM probably involves avoiding early sport specialization or reducing the axial loading, as the development of PCM is strongly associated with cutting and impingement sport practicing (eg, soccer, basketball, ice hockey) and higher levels of physical activity during skeletal growth. However, primary prevention presents several significant challenges.^23^ First, while PCM develops during adolescence, intra-articular damages related to PCM may not occur until young adulthood.^8,21^ A large proportion of individuals with PCM remain asymptomatic during adolescence. This raises concerns about overdiagnosis and unnecessary interventions, as not all cases will progress to FAIS and hip osteoarthritis. Second, recommendations to reduce external loading during adolescence may conflict with guidelines promoting regular physical activity for overall health and development. Third, the specific types and volume of loading contribute to the development of PCM remain unclear. Without this knowledge, it may be premature to implement primary prevention strategies. Future longitudinal studies are needed to identify which individuals are at higher risk for symptomatic progression or hip osteoarthritis and to develop targeted preventive strategies accordingly.

The major strengths of our study include a multiethnic population-based design with a large sample size in early adolescents and the use of automated measures of the alpha angle to define PCM. Our study also had limitations. First, causal inferences cannot be drawn from the cross-sectional design of this study. Second, we obtained DXA scans of only the right hip, which underestimates the presence of PCM as it can be present in the left hip.Third, our study provided only the AP view of the right hip, which might result in an underestimation of the prevalence of PCM as compared with the Dunn view and 3-dimensional imaging (eg, magnetic resonance imaging) because cam morphology is a 3-dimensional entity that typically develops at the anterolateral femoral head-neck junction of the hip.Fourth, using the alpha angle as a continuous outcome may reduce its clinical relevance. In particular, small increments of increase in low alpha angles (eg, between 35° and 45°) may not be meaningful in clinical practice and are unlikely to be associated with FAIS and hip osteoarthritis. Fifth, by focusing only on the self-reported sport types and the frequency of physical activity, our study may not capture the full effect of physical activity on PCM. Using objective measurement tools in future studies could provide a more comprehensive, objective assessment of physical activity and its relationship to PCM than questionnaires. Another limitation is a relatively high percentage of missing data on sport types (17.7%) and physical activity frequency (31.7%). This may lead to the potential of biased estimates, although we utilized MICE to handle the missingness. Moreover, our study captures only a snapshot of early adolescence, and the results may not be generalized to young adults. Because PCM develops over time in adolescence, additional participants could still develop PCM in the future. Therefore, these findings should be generalized cautiously beyond this age group and be followed up in the future. Last, although our study considered multiple associated factors, other important factors—such as acetabular morphology, femoral torsion, and other factors contributing to hip instability—were not included in the current analysis. These factors may influence PCM in early adolescents and warrant further investigation in future studies.

## Conclusion

Our cross-sectional analyses from the Generation R study found a PCM prevalence of 3.4% and associations of male sex, closed proximal femoral physis, and underweight with the presence of PCM in early adolescents. Male sex, closed proximal femoral physis, cutting sport type, overweight, and obesity were associated with increased alpha angle in early adolescents from the general population. These findings could inform potential primary prevention strategies for PCM in the general population during critical growth periods. Future longitudinal studies are required to explore the causality of these associations.

## Appendix

**Table A1:** Observed and Imputed Characteristics of Participants (N = 4477)*^*a*^*

	Data Set, No. (%)	
Characteristic	Observed	Imputed
Age, y	13.5 (13.1-14.6)*^*b*^*	NA
Sex		
Female	2311 (51.6)	NA
Male	2166 (48.4)	NA
Ethnicity		
European	3298 (75.3)	3368 (75.2)
African	693 (15.8)	713 (15.9)
Asian	257 (5.9)	264 (5.9)
Other	129 (3.0)	132 (3.0)
Proximal femoral physis status		
Open	2626 (58.7)	NA
Closed	1851 (41.3)	NA
Weight categories		
Underweight	468 (10.4)	NA
Normal weight	3280 (73.3)	NA
Overweight	578 (12.9)	NA
Obesity	151 (3.4)	NA
Physical activity frequency		
Low	1004 (32.8)	1526 (34.1)
High	2054 (67.2)	2951 (65.9)
Sport type		
None	598 (16.2)	761 (17.0)
Cutting	1434 (38.9)	1720 (38.4)
Flexibility	483 (13.1)	587 (13.1)
Asymmetric	229 (6.2)	274 (6.1)
Impingement	227 (6.2)	276 (6.2)
Endurance	232 (6.3)	284 (6.4)
Other	34 (0.9)	41 (0.9)
Mixed	448 (12.2)	534 (11.9)
Primary cam morphology	151 (3.4)	NA
Alpha angle, deg	43.4 (35.4-64.0)*^*b*^*	NA

a NA, not applicable.

b Median (2.5th-97.5th percentile).

**Table A2:** Associated Factors of Primary Cam Morphology Among Early Adolescents in the Complete Case Data Set (n = 2874)*^*a*^*

	aOR (95% CI)	*P* Value
Age	1.18 (0.64-2.01)	.572
Sex		
Female	1 [Reference]	
Male	2.09 (1.14-3.88)	.018
Ethnicity		
European	1 [Reference]	
African	0.78 (0.35-1.52)	.496
Asian	0.81 (0.24-2.03)	.688
Other	1.34 (0.32-3.82)	.631
Proximal femoral physis status		
Open	1 [Reference]	
Closed	3.01 (1.62-5.62)	<.001
Weight categories		
Underweight	0.20 (0.03-0.64)	.025
Normal weight	1 [Reference]	
Overweight	0.68 (0.30-1.36)	.317
Obesity	0.45 (0.02-2.17)	.437
Physical activity frequency		
Low	1 [Reference]	
High	1.38 (0.84-2.35)	.219
Sport type		
None	1 [Reference]	
Cutting	1.06 (0.54-2.17)	.879
Flexibility	0.80 (0.32-1.90)	.612
Asymmetric	0.79 (0.25-2.18)	.664
Impingement	0.66 (0.18-1.94)	.482
Endurance	1.20 (0.46-2.96)	.704
Other	4.30 (0.90-15.37)	.038
Mixed	0.90 (0.37-2.13)	.806

a Multivariable logistic regression analysis was based on the completed case data set. aOR, adjusted odds ratio.

**Table A3:** Associated Factors of Increased Alpha Angle Among Early Adolescents in the Complete Case Data Set (n = 2874)*^*a*^*

	Adjusted β Coefficient (95% CI)	*P* Value
Age	–0.12 (–0.92, 0.69)	.776
Sex		
Female	0 [Reference]	
Male	2.88 (2.18, 3.58)	<.001
Ethnicity		
European	0 [Reference]	
African	–0.88 (–1.72, –0.04)	.040
Asian	–0.76 (–1.93, 0.41)	.202
Other	–0.16 (–1.81, 1.49)	.848
Proximal femoral physis status		
Open	0 [Reference]	
Closed	1.32 (0.59, 2.04)	<.001
Weight categories		
Underweight	–1.07 (–1.91, –0.23)	.012
Normal weight	0 [Reference]	
Overweight	0.58 (–0.29, 1.45)	.188
Obesity	1.15 (–0.72, 3.02)	.229
Physical activity frequency		
Low	0 [Reference]	
High	0.36 (–0.24, 0.96)	.241
Sport type		
None	0 [Reference]	
Cutting	0.95 (0.10, 1.80)	.029
Flexibility	–0.14 (–1.15, 0.87)	.791
Asymmetric	0.26 (–0.95, 1.47)	.671
Impingement	–0.19 (–1.44, 1.06)	.765
Endurance	1.08 (–0.13, 2.30)	.080
Other	1.81 (–0.91, 4.54)	.192
Mixed	0.48 (–0.56, 1.52)	.362

a Multivariable linear regression analysis was based on the complete case data set.

**Table A4:** Sensitivity Analyses on Associated Factors of Primary Cam Morphology Among Early Adolescents Excluding Mixed Sports*^*a*^*

	aOR (95% CI)	*P* Value
Age	1.15 (0.71-1.87)	.559
Sex		
Female	1 [Reference]	
Male	2.23 (1.29-3.86)	.004
Ethnicity		
European	1 [Reference]	
African	0.92 (0.52-1.62)	.766
Asian	0.47 (0.14-1.54)	.213
Other	1.20 (0.43-3.37)	.726
Proximal femoral physis status		
Open	1 [Reference]	
Closed	3.30 (1.88-5.78)	<.001
Weight categories		
Underweight	0.26 (0.08-0.84)	.024
Normal weight	1 [Reference]	
Overweight	0.56 (0.27-1.12)	.102
Obesity	1.05 (0.37-3.02)	.921
Physical activity frequency		
Low	1 [Reference]	
High	1.15 (0.70-1.91)	.583
Sport types		
None	1 [Reference]	
Cutting	1.00 (0.57-1.76)	.990
Flexibility	0.55 (0.25-1.19)	.129
Asymmetric	0.71 (0.28-1.81)	.476
Impingement	0.55 (0.20-1.49)	.241
Endurance	1.06 (0.49-2.33)	.875
Other	2.93 (0.79-10.82)	.107

a Multivariable logistic regression analysis was based on the imputed data set and was the pooled result of 40 data sets. aOR, odds ratio.

**Table A5:** Sensitivity Analyses on Associated Factors of Increased Alpha Angle Among Early Adolescents Excluding Mixed Sports*^*a*^*

	Adjusted β Coefficient (95% CI)	*P* Value
Age	0.40 (–0.34, 1.13)	.292
Sex		
Female	0 [Reference]	
Male	2.81 (2.14, 3.48)	<.001
Ethnicity		
European	0 [Reference]	
African	–0.44 (–1.20, 0.31)	.275
Asian	–1.24 (–2.39, –0.09)	.034
Other	0.08 (–1.39, 1.55)	.918
Proximal femoral physis status		
Open	0 [Reference]	
Closed	1.12 (0.42, 1.81)	.002
Weight categories		
Underweight	–1.21 (–2.02, –0.39)	.004
Normal weight	0 [Reference]	
Overweight	0.31 (–0.49, 1.10)	.450
Obesity	2.00 (0.46, 3.54)	.011
Physical activity frequency		
Low	0 [Reference]	
High	0.28 (–0.36, 0.91)	.394
Sport type		
None	0 [Reference]	
Cutting	1.07 (0.31, 1.83)	.006
Flexibility	–0.35 (–1.24, 0.53)	.432
Asymmetric	0.35 (–0.77, 1.47)	.539
Impingement	–0.33 (–1.46, 0.80)	.563
Endurance	0.96 (–0.16, 2.07)	.093
Other	1.01 (–1.49, 3.51)	.428

a Multivariable linear regression analysis was based on the imputed data set and was the pooled result of 40 data sets.
